# Carbohydrate and sleep: An evaluation of putative mechanisms

**DOI:** 10.3389/fnut.2022.933898

**Published:** 2022-09-21

**Authors:** David Benton, Anthony Bloxham, Chantelle Gaylor, Anthony Brennan, Hayley A. Young

**Affiliations:** Department of Psychology, Swansea University, Wales, United Kingdom

**Keywords:** carbohydrate, glucose-sensitive cells, melatonin, serotonin, sleep, sleep and energy homeostasis, tryptophan

## Abstract

Sleep problems are extremely common in industrialized countries and the possibility that diet might be used to improve sleep has been considered. The topic has been reviewed many times, resulting in the frequent suggestion that carbohydrate increases the uptake of tryptophan by the brain, where it is metabolized into serotonin and melatonin, with the suggestion that this improves sleep. An alternative mechanism was proposed based on animal literature that has been largely ignored by those considering diet and sleep. The hypothesis was that, as in the hypothalamus there are glucose-sensing neurons associated with the sleep-wake cycle, we should consider the impact of carbohydrate-induced changes in the level of blood glucose. A meta-analysis found that after consuming a lower amount of carbohydrate, more time was spent in slow-wave sleep (SWS) and less in rapid-eye-movement sleep. As the credibility of alternative mechanisms has tended not to have been critically evaluated, they were considered by examining their biochemical, nutritional, and pharmacological plausibility. Although high carbohydrate consumption can increase the uptake of tryptophan by the brain, it only occurs with such low levels of protein that the mechanism is not relevant to a normal diet. After entering the brain tryptophan is converted to serotonin, a neurotransmitter known to influence so many different aspects of sleep and wakefulness, that it is not reasonable to expect a uniform improvement in sleep. Some serotonin is converted to melatonin, although the exogenous dose of melatonin needed to influence sleep cannot be credibly provided by the diet. This review was registered in the International Prospective Register of Systematic Reviews (CRD42020223560).

## Introduction

Fifty to seventy million Americans have a sleep disorder (1) and in Australia, a problem was found in 33–45% of adults (2). Given the physical and psychological consequences of sleep loss, one way of attempting to improve sleep patterns has been to consider diet, with carbohydrate being the nutrient most commonly suggested to be influential.

To date, when relating diet to sleep, putative mechanisms have proposed that a single nutrient, such as tryptophan or melatonin, will generically improve sleep (3–8). There are three justifications for the present review. Firstly, there is a need to establish whether the presently discussed carbohydrate-related mechanisms are biochemically, nutritionally, and pharmacologically plausible. Secondly, as there are different stages of sleep, the possibility was considered that diet differentially influences different stages, as to date there has been a tendency to treat sleep as a homogeneous phenomenon. Single measures, such as sleep latency, sleep duration, or a subjective assessment of quality, have been presented as demonstrating a general improvement. Finally, given that problems were established, an alternative approach, based on glucose levels, was outlined.

Sleep proceeds through a series of cycles in which non-Rapid Eye Movement sleep (NREM) is followed by Rapid Eye Movement sleep (REM) (9). NREM has three stages, labeled N1, N2, and N3. Sleep is light during the N1 stage of NREM as it is the transition between wakefulness and sleep. In the N2 stage of NREM brain waves become slower as you begin to sleep. On entering the N3 stage, slow delta waves occur that are associated with so-called deep or slow-wave sleep (SWS). REM, also called paradoxical sleep, then follows. It is characterized by rapid movements of the eyes, a low muscle tone, and a pattern of brain waves similar to a waking state. Each cycle of NREM and REM lasts on average from 90 to 110 min, with SWS occurring to a greater extent in the first half of the night, whereas the time spent in REM, increases as the night progresses.

It is important that these stages have different functions as if it proves possible to use diet to selectively enhance a stage of sleep, particular aspects of functioning may be enhanced. Dreaming occurs during both NREM and REM sleep, although during REM they are more vivid and memorable. In contrast, during NREM, dreams are mundane (10). Two suggested functions for dreams are that they are involved in the consolidation of memories and the processing of emotions. Whereas both REM and NREM have a role in memory, the roles differ (11). SWS is associated with the generation of Adenosine Triphosphate (ATP), cell regeneration and bodily repair (12–14).

A two-process model of the regulation of the sleep-wake cycle has been proposed (15). Process S reflects homeostatic pressures that increase the need to sleep, a reflection of how long you have been awake. Process C is regulated by the circadian clock, which depends on the phase of the body’s circadian rhythm. When considering diet and sleep, an obvious place to start is the hypothalamus as there are glucose-sensitive neurons (16) containing neuropeptide-producing neurons that are important in regulating sleep.

### Brain functioning

The brain is metabolically active and although it is only 2% of body weight it is responsible for 20% of basal metabolic rate (17). Yet the brain relies on a continuous supply of glucose (17), an observation that is important as the different stages of sleep vary greatly in their neuronal activity and hence their demand for glucose. How then does the supply of glucose to the brain relate to the changing demands for energy that characterize the different stages of sleep? Are these influenced by diet? This perspective differs from the traditional assumption that carbohydrate consumption is a major cause of the uptake of tryptophan by the brain (18), increasing the synthesis of serotonin and melatonin, with consequences for sleep (3–8).

Initially, the mechanisms most commonly used to explain the influence of carbohydrate were reviewed; in particular, the possible roles of tryptophan, serotonin, and melatonin. As these mechanisms were found wanting, an alternative was suggested that draws on animal literature relevant to diet. Do glucose-sensing neurons in the hypothalamus respond to dietary-induced changes in the levels of blood glucose to influence both glucose homeostasis and sleep (19–25).

## Mechanisms by which carbohydrate may affect sleep

### Carbohydrate, glucose, and the brain

Within minutes of eating a meal, the breakdown of carbohydrate increases the levels of blood glucose, to an extent that reflects the amount and nature of the carbohydrate. The greater the rise in glucose, the more that insulin is released from the pancreas, and the faster levels decline.

The brain differs from the rest of the body in that under normal circumstances, it depends almost entirely on glucose as its source of fuel (17). A rise in activity in an area of the brain is associated with increased neuronal activity and the demand for glucose. In the present context, REM is associated with intense neuronal activity (9). Yet the brain has only a limited store of glucose, and if it is not replaced from the blood, brain functioning is compromised in 5–10 min (17): thinking is confused, vision blurred, and speech slurred. Therefore, the body attempts to keep the level of blood glucose within a prescribed range.

When glucose levels are too high, insulin removes it from the blood. When the levels are too low, the hormone glucagon releases stored glucose from the liver. After a meal the carbohydrate consumed increases blood glucose levels, reaching a zenith after 20–30 min, after which in non-diabetics it falls. It is possible that the evolution of these hormonal mechanisms reflects the need to maintain blood glucose within the range necessary for brain functioning.

As the activity of the brain varies throughout the night (9), the supply and use of glucose will differ with the stage of sleep. Carbohydrate is the major source of blood glucose, and its consumption has repeatedly been related to sleep, with the most commonly proposed mechanisms being changes in the levels of brain tryptophan, serotonin, or melatonin (3–8). These putative mechanisms are therefore considered.

### Tryptophan

The most common mechanism mentioned when considering the influence of carbohydrate on sleep, is that it facilitates the uptake of tryptophan by the brain. The review of Peuhkuri et al. (4) concluded that “…. foods impacting the availability of tryptophan, as well as the synthesis of serotonin and melatonin, may be the most helpful in promoting sleep.” The synthesis of serotonin depends on the provision of tryptophan, which is converted by tryptophan hydroxylase, and amino acid decarboxylase, to serotonin. As the rate-limiting step is tryptophan hydroxylase, an enzyme not normally saturated, an increased provision of tryptophan raises the level of brain serotonin (26).

**FIGURE 1:**
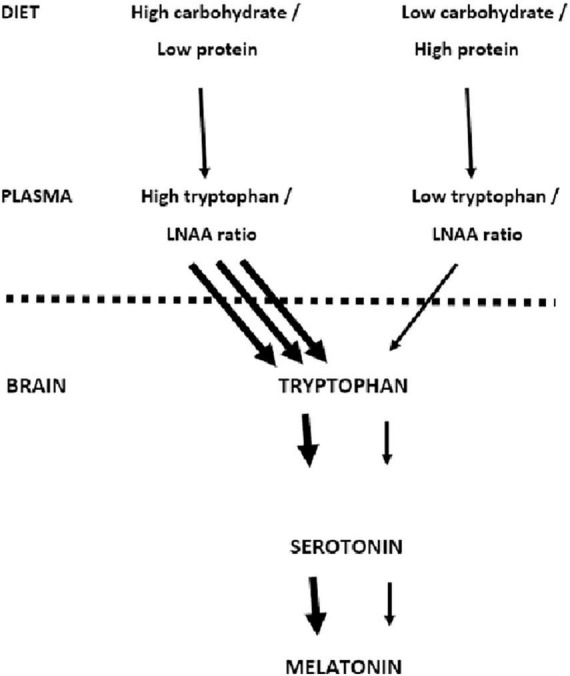
Dietary carbohydrate and the uptake of tryptophan by the brain. The mechanism proposed by Fernstrom and Wurtman (18) is outlined. The suggestion is that a high carbohydrate meal increases the uptake of tryptophan by the brain increasing the synthesis of serotonin and melatonin.

A particular mechanism has been discussed (Figure 1). Carbohydrate-induced increases in blood glucose have been said to increase the uptake of tryptophan by the brain (18), increasing the synthesis of serotonin and melatonin. For example, when Binks et al. (8) reviewed the influence of diet on sleep, they proposed that a major factor was the ability to vary the activity of brain serotonin and hence increase melatonin production. Fundamental to this suggestion is the work of Fernstrom and Wurtman (18). Consuming carbohydrate rapidly increases the levels of blood glucose with a consequent release of insulin, which causes muscle to take up Long Chain Neutral Amino Acids (LNAA—tyrosine, phenylalanine, leucine, isoleucine, valine). An exception is tryptophan which binds to albumin and remains in the blood.

Although normally all LNAAs compete for access to the same brain transporter molecule (13), tryptophan is present in smaller amounts than other LNAA and therefore relatively less enters the brain (Figure 1). Thus after the consumption of a meal that is exclusively carbohydrate, the insulin-induced uptake of LNNA by muscle results in there being relatively more tryptophan in the blood. Consequently more enters the brain (18) where it said that it is metabolized into serotonin and melatonin; in this way facilitating sleep.

The important question is what role, if any, does the Fernstrom and Wurtman (18) mechanism play in a normal diet? Is it realistic to expect that a normal variation in carbohydrate consumption will influence plasma levels of tryptophan, and hence its uptake by the brain? The key point is not the concentration of plasma tryptophan, but rather the ratio between tryptophan and the total of all LNAA. Benton and Donohoe (27) looked at 30 human studies that had examined meals differing in the percentage of energy coming from protein (Table 1). There was some support for the Fernstrom and Wurtman (18) theory. If a high level of carbohydrate was provided, and protein offered less than 2% of the calories, the proportion of LNAA made up by tryptophan was significantly greater. The majority of such studies provided a drink that was 100% carbohydrate and contained no protein.

**TABLE 1:** The influence of protein in a meal on the ratio tryptophan/total LNAA.

Percentage of calories from protein	The ratio of tryptophan/LNAA as % baseline
<2%	123%
4%	109%
5–10%	101%
12–19%	96%
20–49%	75%
>50%	67%

Thirty studies are summarized that related the amount of protein consumed, to the resulting ratio between tryptophan and other Long-chain Neutral Amino Acids (LNAA) expressed as a percentage of baseline values. The data are from Benton and Donohoe (27).

Such is the belief that, when it comes to sleep, the uptake of tryptophan is responsible for the effects of carbohydrate (3–8), any suggestion that this mechanism is unlikely to be relevant will be viewed with skepticism. It should, however, be understood that the present argument is based on the initial reports of Fernstrom and Wurtman who developed the hypothesis (18). In research using rats, commissioned by NASA (28), he compared the response to different combinations of carbohydrate and protein, in meals that were up to 20% protein, and up to 75% carbohydrate.

When a meal contained 70–75% of carbohydrate, the ratio between plasma tryptophan and other LNAA increased; something not observed with 25% carbohydrate. However, adding 5% of the protein casein to 70% carbohydrate, fully blocked the increase in the tryptophan ratio. The pattern was the same as observed in human studies (27) (Table 1). Yokogoshi and Wurtman suggested that the mechanism was “probably by contributing much larger quantities of the other LNAA than of tryptophan to the blood” (28). As protein provides more of the LNAA other than tryptophan, the entry of the latter into the brain is reduced.

Those wedded to tryptophan having a role in sleep, need to compare the impact of realistic meals. Do these meals increase the levels of tryptophan relative to other LNAA? Does the availability of tryptophan produce differences in sleep architecture? As there will be individual differences in the response to the same meal, do individual differences in the sleep response to a particular meal, depend on the extent to which the levels of tryptophan change?

An example is offered by Wurtman (28) who contrasted unlikely meals that were high carbohydrate (Carbohydrate (C): 80%: Protein (P) 6%; Fat (F):14%) or high protein meal (C:17%; P:53%; F:30%). Four hours after the consumption of the carbohydrate meal the ratio of Tryptophan/LNAA increased from baseline by 10.2%. However, the ratio decreased by 34.8% after the high protein meal. Although the emphasis was placed on the increased ratio following carbohydrate, it was less than would have resulted if there had been no protein (27, 28). A more informative conclusion would have been that higher intakes of protein, which are typical of the majority of meals in industrialized countries, reduce the availability of tryptophan. To increase the ratio you need a meal unlike those that are usually consumed (29).

Under laboratory conditions when 100% carbohydrate has been consumed, the provision of tryptophan increases (27), although with *ad libitum* feeding it is a phenomenon that will occur rarely and only with contrived meals. In the USA the average energy intake from protein is 16.3% for men and 15.8% for women. The average intake of carbohydrate is 46.4% for men and 48.2% for women (30). The acceptable ranges of macronutrient consumption are 10–35% of your daily calories from protein and 45 to 65% from carbohydrate (29). Such data illustrate that there would need to be a substantial change in the diet of almost all of the population before the intake of protein was low enough to influence sleep. These data illustrate that the meals, used by Wurtman et al. as an exemplar (31), are vastly different from the normal diet and if consumed regularly will result in an unbalanced diet.

In addition, an almost exclusively carbohydrate meal would have to be eaten shortly after a previous meal had left amino acids in the blood. Tryptophan is an essential amino acid, and therefore needs to be consumed as it cannot be made by the body. Therefore, for the Fernstrom and Wurtman (18) mechanism to be relevant, tryptophan needs to have been provided by a protein-containing meal in the recent past, followed by a subsequent meal with, at the most, an extremely low amount of protein. Amino acids, as such, are not stored by the body but are found in the blood in low amounts, but only for up to 24 h after a meal. Many are converted to glucose by the liver.

Afaghi et al. (32) tested the tryptophan hypothesis by conducting a study that varied the glycemic load (GL: the amount of glucose released) of an evening meal, while maintaining a consistent macro-nutrient profile (C: 90.4%; P: 8%; F: 1.6%). The GL was varied by providing rice with different glycemic indices (50 or 109), resulting in a GL of either 81.3 or 175. Polysomnography, however, produced no differences in sleep architecture, although the sleep onset time was shorter after the higher GL meal. Although the glucose profile differed depending on the meal, tryptophan levels were not assessed. However, given the amount of protein eaten, the probability was that the tryptophan levels had not increased (Table 1). In addition levels of a melatonin metabolite had not risen, suggesting that the levels of brain tryptophan had not increased.

It is difficult to avoid the conclusion that it is much easier to reduce rather than increase the uptake of tryptophan by the brain. As such, it is unlikely that tryptophan uptake, and the creation of serotonin, will be increased by the diet eaten in industrialized countries. So-called high carbohydrate foods, such as potatoes or bread, provide 10 and 15% of calories as protein; levels that will either have no influence or will decrease the provision of tryptophan.

Even if it was possible to create a highly prescribed meal that increased brain tryptophan uptake, the expectation that this will encourage sleep offers too simple a perspective. The pharmacological administration of tryptophan in the absence of other LNAA will inevitably increase the ratio of tryptophan to other LNAA in the blood: therefore more tryptophan will enter the brain (26). However, when tryptophan is consumed as a pharmacological supplement, with a dose of 1–15 g, there are inconsistent reports that sleep is facilitated (33). This inconsistency occurs with changes in tryptophan levels much greater than are possible with diet, although pharmacological doses will inevitably increase the synthesis of serotonin (26).

### Serotonin

Even if the synthesis of serotonin did rise following an increased uptake of tryptophan, it is unclear why it would be thought to have a uniformly beneficial impact on sleep. Serotonergic neurons originate in the dorsal and median raphé nuclei of the brain stem: they project throughout the brain and are involved with many functions including circadian rhythms (34). Increased levels of serotonin do not, however, have an inevitable beneficial influence on sleep.

The activity of the raphé nucleus is lower during NREM sleep and suppressed during REM (35). In the brain, selective serotonin reuptake inhibitors increase serotonin at the synapse and are known to inhibit REM (36). However, a systematic review concluded that selective serotonin reuptake inhibitors, rather than improving sleep, decrease the quality of sleep and increase insomnia (37).

A brief summary of a complex field is that the role of serotonin in sleep/wakefulness reflects the activity of a range of sub-types of serotoninergic receptors, involved in the control of different aspects of sleep. These roles involve complex interactions with other pathways that are mediated by a range of other neurotransmitters (38). Serotonin has a role in various stages of sleep: it can help to maintain sleep but also helps you stay awake. There is little reason to suggest that if a carbohydrate-induced increase in serotonin was to occur, serotonin as such, would generally benefit sleep (38).

### Melatonin

Sanlier and Sabuncular (6) proposed that when looking for food to promote sleep, those containing tryptophan were promising candidates, as it was the precursor of both serotonin and melatonin. An important role for serotonin is that it is converted by N-acetyl transferase and 5-hydroxyindole-O-methyltransferase into melatonin, with consequences for sleep (39). However, although it is unlikely that carbohydrate consumption will increase the levels of melatonin in the brain, even if it did there are additional reasons to question the suggestion that such a mechanism would facilitate sleep.

The pineal gland, found in the roof of the third ventricle of the brain, controls the sleep-wake cycle. Signals from the eyes, in response to light and darkness, induce the formation and release of melatonin, which helps in falling asleep. Melatonin is produced at night (40) and plays a basic role in regulating the biological clock. Serotonin, as such, is more concerned with wakefulness, the onset of sleep, and in particular suppressing REM (38). A particular issue is that when serotonin acts as the precursor of melatonin this depends upon darkness, as both sunlight and artificial light inhibit the synthesis of melatonin (41). Even if a high intake of carbohydrate did increase the levels of brain tryptophan, melatonin would only be synthesized when it was dark.

Melatonin is used as a supplement to treat insomnia or jet lag, and a review found evidence of reduced sleep latency and an increased time sleeping, although there was no consensus that these changes were clinically meaningful (42). The doses used were between 0.5 and 5 mg. It is, however, difficult to demonstrate that the impact of any dietary-induced increase in brain melatonin synthesis has consequences for sleep. The production of melatonin is greatest in the middle of the dark period, as short-wavelength blue light effectively suppresses its production (43). After it has been synthesized in the pineal gland, melatonin is not stored but rather released into the blood, where it has a half-life of 40–60 min. The half-life of an exogenous source is between 10 and 60 min (43).

Additional information comes from the use of foods naturally high in melatonin, such as tart cherries. Pereira et al. (5) considered randomized, placebo-controlled trials with foods containing melatonin, and concluded that these food sources could help to prevent and treat sleep disorders. As an example, tart cherries (13 nano-grams melatonin/gram cherry), have been found to modestly influence sleep, but not to an extent that is clinically beneficial (44). However, to acquire 0.5 mg melatonin, the minimal active exogenous dose (42), 38 kg of tart cherries would need to have been eaten. It is improbable that a clinically significant dose has ever been consumed.

Howatson et al. (45) gave tart cherry juice for 7 days and an increased level of a melatonin metabolite was associated with both the time spent sleeping and sleep efficiency. It was concluded that exogenous melatonin can improve sleep. There are reasons to question this conclusion as during darkness melatonin is released when you sleep, irrespective of what you have eaten. Sigurdardottir et al. (46) reported that the levels of melatonin metabolites in urine were related to the quality of sleep, so those with sleep problems had lower levels in the morning. As Howatson et al. (45) found that sleep was longer when taking cherry juice, it is plausible that what was being measured was the quality or length of sleep, rather than the melatonin in the drink. As consumption was equivalent to eating about 200 cherries this would have provided 85 m-g of melatonin. This intake is about a sixth of the lowest levels of exogenous melatonin that has been found to influence sleep (500–1,000 mg) (42), making it unlikely that a pharmacological dose had been consumed. There is a need to consider alternative ways in which cherries might facilitate sleep. One possibility, that has been largely ignored, is a potential role for the GABA (A) receptor, the mechanism by which the three generations of hypnotic drugs all act. Cherries are rich in flavonoids, that have been said to act as GABA-benzodiazepine receptor ligands (47).

In summary, it may well be that any suggestion that the “sleep hormone” melatonin does not improve sleep will seem counter-intuitive. After all, there is no doubt that melatonin plays a central role in the sleep-wake cycle, and as it becomes dark it is released by the body to promote sleep. However, after reviewing relevant studies, the American Academy of Sleep Medicine concluded that there was insufficient evidence to recommend using either tryptophan or melatonin to reduce insomnia (48). As they were talking about pharmacological doses, an order of magnitude greater than could possibly result from the diet, this is a good reason to suggest that the use of food is unlikely to be successful. A fundamental reason is it is improbable that any normal diet will be able to increase the levels of brain tryptophan (27, 28). In addition, to be successful a melatonin-containing meal would need to be consumed in a dark room (41), in improbably large quantities (42), the hour before going to bed (43).

In any event, it will prove difficult to demonstrate a role for melatonin, as it is released from the brain while we sleep. If dietary manipulation improves sleep, we would not be able to establish whether any increase in the release of melatonin is caused by diet, rather than resulting from improved sleep that was induced by some other mechanism.

## Blood glucose and sleep

As the proposal that exogenous melatonin, serotonin, or tryptophan influence sleep does not stand examination, we need an alternative. A potential approach is provided by an extensive physiological literature that has been largely ignored by those studying the influence of diet. It was therefore considered whether it was the influence of carbohydrate on blood glucose that affects sleep.

Burdakov and Adamantidis (16) asked a basic question: “do nutrients acutely change the firing rates of neurons implicated in sleep control, and does this alter sleep”? If we are to look for such a mechanism an obvious starting point is glucose, the basic fuel of the brain. A potential approach is to consider areas of the brain that control sleep and, given the inevitable relationship between carbohydrate consumption and increases in blood glucose, to look for possible interactions.

### Sleep and energy homeostasis

To establish the influence of sleep on glucose regulation, a constant infusion of glucose has been used to prevent the endogenous production of glucose, and in this way monitor changes in the brain’s use of its basic fuel. During the early part of the night the levels of glucose increase by about twenty percent, suggesting a lower demand for glucose by the brain (49). The use of positron emission tomography (PET) similarly found a reduction of about 11% in the use of glucose by the whole brain during NREM (50). However, a return to baseline glucose levels occurs late in the night (49, 51). There appears to be an association between the control of glucose and sleep, as when sleep occurred during the day there was also an increase in glucose levels (52).

As the expenditure and intake of energy need to be balanced, it is controlled by the brain with the hypothalamus exerting an important role (53). Although the way that the hypothalamus senses glucose has proved difficult to establish, more recently it has been suggested that there is strong support for “a role for glucose signaling in regulating energy balance, glucose homeostasis, and food-induced reward” (54).

Circulating levels of glucose can be sensed by neurons in the arcuate and ventromedial nuclei of the hypothalamus (55, 56), and using functional MRI Varin et al. (21) monitored the activity of hypothalamic neurons and found, when humans consumed glucose, there was a persistent decrease in the BOLD signal. An intravenous administration produced a modest and transient decline in the signal. However, when supplied as a drink, a larger and longer-lasting reduction in the BOLD signal occurred. It was suggested that signals from the gastrointestinal tract play an important role in the response of the hypothalamus. The sensing of glucose by the ventromedial hypothalamus has been suggested to play a critical role in energy balance, with “glucose-excited” and “glucose-inhibited” neurons firing in the ambient glucose range of 1–5 mM (57).

For many years it has been accepted that the hypothalamus has antagonistic centers that promote sleep or being awake (58, 59). Varin (21) found cells in the ventrolateral preoptic nucleus that were active during sleep and mediated the onset of NREM sleep. Sherin et al. (19) recorded the activity of single cells in the brain and found a high number in the lateral hypothalamus, whose activity was influenced by glucose concentration. The rate of firing of such cells could be either increased or decreased, often within seconds. More specifically, Oomura et al. (20) reported that the concentration of glucose influenced the activity of seventy-three percent of neurons in the ventrolateral preoptic nucleus, an area that promotes sleep: they proposed that in this way glucose levels contribute to the onset of SWS. The ventrolateral preoptic nucleus is said to promote sleep by inhibiting the ascending reticular arousal system; thus having a role in generating and maintaining SWS (60).

### Orexin and melanin-concentrating hormone neurons

In the lateral hypothalamus of the mouse, the level of extracellular glucose was found to influence orexin (hypocretin) neurons that promote wakefulness. Both optogenetic stimulation of orexin neurons, and injecting orexin into the brain are associated with wakefulness. These neurons are active when awake and inactive when asleep (61). Sleep involves switching between non-REM and REM sleep, changes mediated by “REM-on” and “REM-off” neurons in the brain stem (61), with glucose-sensitive orexin neurons having a key role as they project to the locus coeruleus, a location of many “REM-OFF” neurons (62). Orexinergic neurons are active when awake but silent during REM and NREM.

In contrast, melanin-concentrating hormone neurons promote sleep and again are excited by glucose. These neurons are silent when awake, but active when asleep, particularly during REM (61, 63). In rodents, the optogenetic stimulation of melanin-concentrating hormone neurons induced sleep, reduced the time awake, and increased both NREM and REM (64). Although basic research has involved the study of animals, in humans, orexin was associated with being awake, whereas melanin-concentrating hormone was found when asleep (65).

The impact of the above mechanisms will be modulated by the stage of the circadian rhythm. The SCN, the pacemaker for circadian rhythms (15), projects to the lateral hypothalamus (66) which contains wake-promoting orexin neurons that have an important role in regulating the sleep/wake state, and the transition between sleep stages (67).

There is some suggestion that the SCN can detect aspects of metabolic status (68). In slices of this area of the hamster’s brain, a concentration of 20 mM rather than 10 mM glucose, advanced the time when there was peak neuronal firing: an effect obtained with glucose but not sucrose. In contrast, there was a slight delay in the peak firing rate with 5 mM glucose (25). Ultimately the SCN is only one factor that influences the sleep-wake cycle and the time since sleeping is particularly important (15).

The SCN has two oscillators, one associated with vasoactive intestinal peptide, and the other with arginine vasopressin. It was reported that the latter oscillator acts as a glucose sensor, while the former remains synchronized to the light-dark cycle (69). A possibility to be explored is that meals with higher levels of carbohydrates increase the concentration of glucose in the brain, causing the SCN to slightly advance the circadian rhythm, with the consequence that REM will occur earlier in the night, resulting in a reduced incidence of SWS.

Adamantidis and De Lecea (23) noted that shared hypothalamic circuits mediate both sleep and the response to energy imbalance, making the relationship between sleep and the provision of energy an appropriate starting point for research. Burdakov and Adamantidis (16) proposed a model where lower levels of glucose at the lateral hypothalamus increased wakefulness and food-seeking behavior: a response associated with increased activity of orexin and lower activity of melanin-concentrating hormone neurons. The reverse was said to result from higher levels of glucose, sleep was facilitated and food-seeking behavior was reduced. In this instance, the activity of orexin neurons was switched off, whereas melanin-concentrating neurons were stimulated.

These conclusions have in part resulted from *in vitro* studies of tissue samples, where, in a dose-dependent manner, neurons responded to different concentrations of glucose (69). If these findings are extrapolated to the living organism, then glucose concentrations will vary continuously, depending on the supply of glucose and physiological demands. It is a reasonable suggestion that there may be a response, not to a precise level, but rather to rising or falling levels of glucose, with consequent changes in the rate of neuronal firing. If so, after a high carbohydrate meal, there will be a rapid increase in blood glucose that will stimulate insulin release, subsequently producing a rapid fall. These falling values will send the message that energy provision is declining, predisposing the individual to be awake to look for food. With a lower carbohydrate meal, the rise in blood glucose will be less and slower. The subsequent fall will be less pronounced, reducing the activity of orexin, and increasing the activity of melanin-concentrating hormone neurons. The response to these mechanisms will, however, depend on the phase of the circadian cycle.

### Influence of carbohydrate on stages of sleep

As there is increasing physiological evidence that neurons, that are sensitive to the level of glucose, play a role in sleep (19–25), a reasonable question is whether meals that differ in the ability to provide blood glucose are influential.

Using polysomnography there have been several reports that the level of carbohydrate in the diet affects sleep architecture. For example, a high carbohydrate meal reduced stage one NREM and increased REM sleep during the first half of the night (68). Low levels of carbohydrate increased the time before REM first appeared (70), increased the percentage of SWS, and reduced the percentage of REM (71). Consuming less carbohydrate resulted in more SWS during the first sleep cycle (72). Those consuming a low carbohydrate drink before bed were less aroused during the night, and reported better sleep quality (73). When following a low carbohydrate diet there was more SWS, whereas a high carbohydrate diet was associated with more REM (74). A study found that both a high intake of saturated fat and carbohydrates was associated with more SWS. In addition, a higher consumption of fiber was associated with more SWS although it remains to be considered whether the mechanism by which fiber increases SWS is slowing the release of glucose (75).

There have also been studies that have assessed sleep using actigraphy. When participants consumed high-protein, high-fat, or a high-carbohydrate diet, it was found that episodes of waking and sleep latencies differed. High-protein diets produced fewer episodes of waking, but in contrast, a high-carbohydrate diet produced a shorter sleep latency (76). Using a similar approach, a high-fat diet was associated with reporting a significantly better quality of sleep, although relevant in the present context, this was the diet with the lowest amount of carbohydrate (25% of energy) (77). A study of children aged 19 months compared the influence of consuming milk, with carbohydrates with either a high or low glycemic index, although it did not influence sleep when assessed using actigraphy (78).

A prospective study of postmenopausal women consuming a diet with a high glycemic load, over 3 years, reported a greater incidence of insomnia. Greater consumption of added sugars, starch, and refined grains was associated with more frequent insomnia and a higher fiber intake with less frequent insomnia (79). However, an impression created is that when examining the influence of diet we need to be using polysomnography, as actigraphy does not give the necessary detail of the stages of sleep.

A meta-analysis, related carbohydrate consumption to the amount of time spent in different stages of sleep (80). It was found that the amount of carbohydrate consumed influenced sleep architecture: a higher intake was associated with less SWS and more REM. The findings were discussed in terms of mechanisms that are traditionally considered, such as tryptophan uptake and the release of gut hormones. It was, however, suggested that higher levels of glucose may be associated with REM, as a higher level of neural activity requires additional energy. However, although such an observation may explain why it happens, it does not propose the mechanism by which it happens.

The above meta-analysis (80) was published while this review was being prepared, although it had always been the intention to perform a meta-analysis as part of the manuscript. To further the aim of this paper a more specific question was asked. In particular, did the impact of carbohydrate on sleep depend on the percentage of energy supplied as protein? Was it only influential when levels of protein are low (Table 1), resulting in the possibility that an increased uptake-up of tryptophan by the brain had influenced sleep (27, 28).

A meta-analysis, the details of which are given as Supplementary Information, examined the effect of meals on sleep. The effects of consuming a higher or lower percentage of energy as carbohydrate, prior to recording the sleep pattern using polysomnography, were examined. When REM was considered the outcome reached significance [RR of −0.47 (95% CI −0.87 to −0.07), *p* = 0.02; *I*^2^ 0%]: a lower consumption of carbohydrate was associated with a shorter duration of REM. With SWS, again the outcome was significant [RR of 0.47 (95% CI 0.06–0.88), *p* = 0.02; *I*^2^ 0%); a lower intake of carbohydrate was associated with a greater incidence of SWS. When sleep onset was considered, a lower intake of carbohydrate was associated with a shorter time before falling asleep [RR of −0.49 (95% CI −0.92 to −0.05), *p* = 0.03; *I*^2^ 0%). There was similarly a trend for better sleep efficiency to be associated with a lower intake of carbohydrate [RR of 0.87 (95% CI −0.02 to 1.76), *p* = 0.07; *I*^2^ 0%].

The nature of the meals responsible for these effects on sleep is reported as Supplementary Table 1. These significant effects on sleep architecture were found in studies that had between 4 and 26% of the energy provided as protein. In fact, there was only one study that had less than 10% of energy as protein, the point at which the proportion of LNAAs made up by tryptophan begins to increase (27, 28). This finding suggests that these effects of carbohydrates on sleep did not depend on an increased provision of tryptophan, and therefore would not lead to an increased synthesis of serotonin and melatonin.

## Discussion

It is attractive to look for simple answers to complex questions. However, with human behavior or physiology, it is an approach that is almost inevitably going to mislead. As an example, seeing tryptophan and melatonin as a way of improving sleep has failed to reflect the complexity of the situation. Similarly, we should not see changes in carbohydrate and blood glucose as the new “silver bullet”: it is important to place the presently discussed mechanism in a broad context.

There was a pattern of a lower carbohydrate intake being associated with more SWS, a pattern associated with a wide range of experimental designs; with evening meals, a pre-sleep snack, or an experimentally produced daily diet (Supplementary Figure 2). The calorie intake in the included studies (68, 70–73, 76) varied greatly without systematically influencing the phenomenon. Thus, the response reflected the relative rather than the absolute level of carbohydrate intake; a finding compatible with the effect reflecting relative differences, rather than the absolute levels of blood glucose. Varying the carbohydrate in meals, which would inevitably result in different levels of blood glucose, produced changes in sleep consistent with previous findings that reported that changing glucose concentrations influenced the firing rate of a high percentage of cells in various areas of the hypothalamus (21, 56, 57). The consumption of glucose has been found to reduce activity in the hypothalamus (21); findings consistent with the conclusion of Burdakov and Adamantidis (16) that “the wake-promoting activity of orexin neurons would be inhibited by a rise in glucose and stimulated by a fall in glucose” and that “glucose-induced excitation of melanin-concentrating hormone neurons may promote sleep and suppress energy expenditure.”

However, though there are interesting parallels between the dietary and neurophysiological data, there is a need for future studies to vary the diet and monitor glucose changes, sleep patterns, and the activity in the hypothalamus. The possibility of a causal association needs to be explored.

However, inevitably there are limitations, and although a way forward has been proposed, it is the outline of an approach rather than a fully demonstrated mechanism. Previously, at least implicitly, a pharmacological approach has been taken; that is attempts have been made to identify a particular nutrient or food item with somnolent properties. It is, however, questionable whether this is a reasonable expectation, as both the control of sleep and the nature of diet are complex. A single change in one aspect of the diet is unlikely, by itself, to benefit all aspects of sleep.

Although the carbohydrate in a meal has a direct influence on the level of blood glucose, diet is multifaceted, and one nutrient should rarely be considered in isolation. Although to date carbohydrate and protein have been discussed, it should not be forgotten that meals also contain fat. Particularly when designing iso-caloric low/high-carbohydrate meals, the energy intake tends to be maintained by varying the fat content. Hence a low/high carbohydrate meal could be described accurately as a high/low-fat meal. In males, a negative association was found between fat intake, sleep efficiency, and REM: whereas in females there was a negative relationship between REM and fat, carbohydrate, and total calorie intake (81). Those who at dinner consumed the highest quartile of fat were more likely to sleep for a short duration (82) and higher consumption of saturated fat or sugar was associated with less SWS (75).

Fat and protein slow the release of glucose into the blood as they influence the glycemic response to carbohydrate by slowing gastric emptying and stimulating the release of insulin (83). When examining the glycemic response to 50 g of glucose, Moghaddam et al. (84) examined the effect of adding either protein or fat. Independently, in a dose-dependent manner both decreased the glycemic response, although protein had three times the effect of fat. However, the influence of protein was modified by dietary fiber, which also slows the glycemic response.

When looking at the response to diet, individual differences in glucose tolerance also need to be considered, as it varies with age, obesity or diabetes (85). For example, diabetes and sleep are closely connected, with at least half of diabetics experiencing insomnia or poor-quality sleep (86). Poor sleep is associated with the development of glucose intolerance in prediabetics (87).

Similarly, we should not ignore the complexity of the mechanisms that control sleep. The focus has been on the hypothalamus, although other brain areas are involved: the thalamus, basal forebrain, pineal gland, and brainstem (88). For example, the thalamus generates SWS (89): the brain stem interacts with the hypothalamus to control the change from being awake to being asleep (90); the amygdala, which processes emotion, is active during REM (91). The hypothalamus is an area where the concentration of glucose influences the rate at which neurons fire (56, 57), however, there are other sub-cortical areas, for example, the brain stem where there are glucose-sensitive neurons (92).

The discussion up to now has been about glucose and carbohydrate, but other mechanisms influence blood glucose levels and energy balance. The hormone ghrelin is released from an empty stomach, after which it is carried by the blood to the hypothalamus where it stimulates food intake and conserves fat (93). It has been called the “hunger hormone,” and as such blood levels are high before a meal and low afterward. Infusing glucose or amino acids directly into the stomach reduces the level of plasma ghrelin (94). As, in addition, it influences glucose tolerance (95) and the sleep-wake cycle (96), ghrelin needs to be considered as potentially influencing any relationship between carbohydrate consumption and the stages of sleep. When ghrelin levels were monitored over 24 h, levels rose in the early hours of the night but decreased in the morning. This secretion of ghrelin in the first hours of sleep correlated with the release of growth hormone that was proposed to facilitate SWS (96).

In many species, including humans, ghrelin has been found to increase the level of blood glucose and worsen glucose tolerance, in part at least a reflection of a reduced release of insulin (95). There are also reports that the ghrelin response differs with the glycemic load. In white but not black American women, a low-glycemic load was associated with a lower level of insulin and blood glucose, but higher levels of ghrelin (97). Racial differences in a glycemic response had been found previously.

When sleep-deprived, the level of ghrelin increases and the level of leptin falls, leading to an increase in hunger (98), indicating that the pre-existing sleep status needs to be controlled in any experimental design. Yet it has been suggested that in humans’ ghrelin is sleep-promoting (99), and a review concluded that ghrelin has a favorable influence on sleep, improving its quality (100). When ghrelin was administered between 2200 and 0100 hours there was increased SWS throughout the night, and REM was less during the middle third of the night. After the administration of ghrelin to mice, there was an increase in NREM although this reaction did not occur if mice lacked growth hormone receptors (101). The release of growth hormone is stimulated by both ghrelin (99) and a rise in glucose (101).

In addition, leptin is produced by adipose tissue and acts at sites in the hypothalamus, where it reduces food intake (49). A major function is to signal that energy stores are reduced and there is a negative energy balance, but there is also an association with sleep. Sleep restriction is associated with lower levels of leptin (102) and hence a tendency to eat more. Even sleep during the day increases the level of leptin. Levels of leptin normally increase during the night and then decline to a minimum in the late afternoon (49).

In mice leptin acutely increased glucose metabolism (103); over a 5 h period an intravenous infusion increased the turnover of glucose, although the levels of glycogen in the liver declined. As a similar result occurred after an intracerebroventricular infusion, it was suggested that the effect was mediated by the brain. In rats, Sinton et al. (104) examined the influence of leptin on the pattern of sleep. The hormone was administered to rats that had been deprived of food for 18 h, or alternatively had received food *ad libitum*. In those who were well-fed leptin reduced REM by about 30%, and increased SWS by about 13%. In contrast, these effects were not shown in the food deprived. These data illustrated that when relating carbohydrate intake and blood glucose to sleep, it is part of a bigger picture and there is a need to consider the levels of leptin, the existing energy balance, and the quality of pre-existing sleep.

When studying the effect of diet, it is natural to direct attention to its composition, although with a given meal other factors come into play. As sleep influences the body’s use of glucose, pre-existing sleep quality will modify the effect of dietary interventions (105). If prevented from sleeping, the levels of glucose and insulin have been found to reach a maximum at the time that sleeping usually began (106), demonstrating that both the stage of the circadian cycle and the pattern of sleeping influence glucose levels and insulin secretion. The time of day a meal is eaten is influential as glucose tolerance varies over the 24-h cycle (107). Thus when the effect of diet is examined, we need to consider both the stage of the circadian rhythm and the time since previously sleeping.

The timing of meals may need to be considered as it has been suggested that food can be a “zeitgeber” (108); the German for time giver or synchronizer, something that acts as a cue to regulate circadian rhythms. Although commonly suggested, a review, after considering the human evidence that is needed to show that food is a circadian zeitgeber, noted that it was limited (108). In addition to the time of day, the sight of food, or even discussing it, causes the mouth to water, and insulin (109) and gastric juices to be released (110), with implications for the rate at which a meal releases glucose, and the rate it later declines.

### The way forward

Although the details have not been fully established, for many years it has been suggested that sleep plays a role in energy balance (111–115), although finding a role for glucose-sensitive neurons in both sleep and energy balance (19–25) suggests these neurons may be part of the mechanism. It is essential for the body to monitor and control the availability of energy and it is proposed that the level of glucose and sleep play a role. Although the above discussion offers a credible basis for the suggestion that changes in blood glucose levels may play a role in controlling the nature of sleep, it is hypothesis-generating rather than confirmatory evidence.

If it can be demonstrated that varying carbohydrate reliably increases SWS (80), this is potentially important as there is a range of disorders associated with a lower incidence of SWS: memory problems (116), neurological problems (117), mood (118), diabetes (119), glucose intolerance (120) and thickening of the arteries (121). However, as these are correlates there is a need to establish a causal relationship, although if demonstrated there would be a dietary means of improving various aspects of functioning. In this context, when studying the impact of diet on sleep, the suggestion of Burdakov and Adamantidis (16) that we should be looking for nutrients that act on neurons implicated in the control of sleep, should be used as a framework. Such an approach should not, however, see carbohydrate as acting alone, as other aspects of diet and physiology influence the levels of blood glucose.

We need to accept that considering a single nutrient is simplistic. Sleep is controlled by multiple and complex mechanisms; sleep is not a unitary phenomenon but rather has various stages, with different modes of control and physiological consequences. Single food items are part of a diet that provides thousands of different molecules, making it unwise to consider one in isolation. As such, there is unlikely to be a simple relationship between all aspects of sleep, and a single food or single nutrient. However, a starting point has been suggested, to which more will be added as further insights become apparent. We should explore the influence of diet on glucose-sensing neurons in the hypothalamus although there is a need to consider the entire diet and the complex mechanisms that control sleep.

## Author contributions

DB, ABl, and HY jointly established the aims of the review. All authors contributed to the analysis, involved in the drafting and interpretation and approved the publication of the manuscript and were accountable for the accuracy and integrity of the work.
