# Supplementary material for: Carbohydrate and sleep: An evaluation of putative mechanisms
Source: Front Nutr. 2022 Sep 21;9:933898. doi: 10.3389/fnut.2022.933898 (PMC9532617; doi:10.3389/fnut.2022.933898)
Supplement: Supplementary file 1 [file Data_Sheet_1.docx]

**SUPPLEMENTARY MATERIAL:**

**META-ANALYSIS OF INFLUENCE OF CARBOHYDRATE ON SLEEP**

The literature was examined to establish dietary patterns associated with sleep.  In particular, attention was directed to the proportions of energy consumed as carbohydrate and protein, as this is critical for any mechanism that relies on the uptake of tryptophan by the brain.  As intense metabolic activity occurs only at some stages of sleep, and as glucose is the main source of energy for the brain, a second aim was to examine whether the amount of carbohydrate consumed differentially influenced stages of sleep that differ in metabolic activity?

The review question was, does the percentage of energy consumed as carbohydrate influence sleep.  In particular, was it only influential when a small percentage of energy came from protein ?

**2. Materials and Methods**

**2.1. Study Design**

A systematic review of peer-reviewed studies, published until July 2021, was performed using the Web of Science, Medline, Cochrane Database and Clinicaltrials.gov.  Studies from any country were included if they had been subject to peer review and published in English.  Review articles were examined for papers that had not been identified previously, and studies quoted by previously identified articles were examined to look for missing studies. The review was registered in the International Prosective Register of Systematic Reviews (PROSPERO) database (CRD42020223560).  The procedure followed the PRISMA guidelines ^1^.

**Population**: All genders and ethnicities were included if they had not exercised on the day of the study;  did not have a history of sleep disorders, for example sleep apnea; there was no report of a diagnosis of a medical complaint that could influence the metabolism of food, for example,  diabetes. Those studies that examined those who had exercised in the day prior to sleep were excluded.

**Intervention**:  Sleep was monitored using polysomnography or actigraphy when food intake, varying in the percentage of energy from carbohydrate, was compared.

**Comparison:**Intervention studies were identified that reported the percentage of energy consumed in the evening prior to sleeping, as protein, carbohydrate, and fat,  and on two occasions had studied the same group when they had consumed carbohydrate in different proportions.

**Outcomes:**Measures obtained from polysomnography included: sleep onset time; sleep efficiency;  rapid eye movement; slow-wave sleep. Alternatively, the measures obtained using actigraphy were sleep onset time; sleep efficiency; duration of sleep.

**Study selection**

The study selection was performed independently, and then by discussion if there was any disagreement,  by two authors (AB, DB). Initially, based on the title and abstract, duplicates, animal studies, and those involving exercise or sleep or medical complaint were excluded.

The full texts were then screened for the inclusion criteria, and relevant details of a study and outcome measures were listed in a table.  Only original research, published in English in a peer-reviewed journal, was retained. Editorials, commentaries, conference abstracts, dissertations, reviews and meta-analyses were excluded, although examined as potential sources of peer-reviewed studies.

The following search terms were used:  Carbohydrate and sleep; Carbohydrate and polysomnography; Carbohydrate and actigraphy; Diet and sleep; Diet and polysomnography; Diet and actigraphy. The a priori primary outcome measures were those obtained from polysomnography that distinguished different sleep stages with measures from actigraphy as secondary outcomes.

**Data Extraction**

Papers were retained if two diets differing in the percentage of calories coming from carbohydrate had been examined, using polysomnography or actigraphy, when data were also available for protein and fat consumption.  The number of studies at each stage is listed in Figure S1.

**
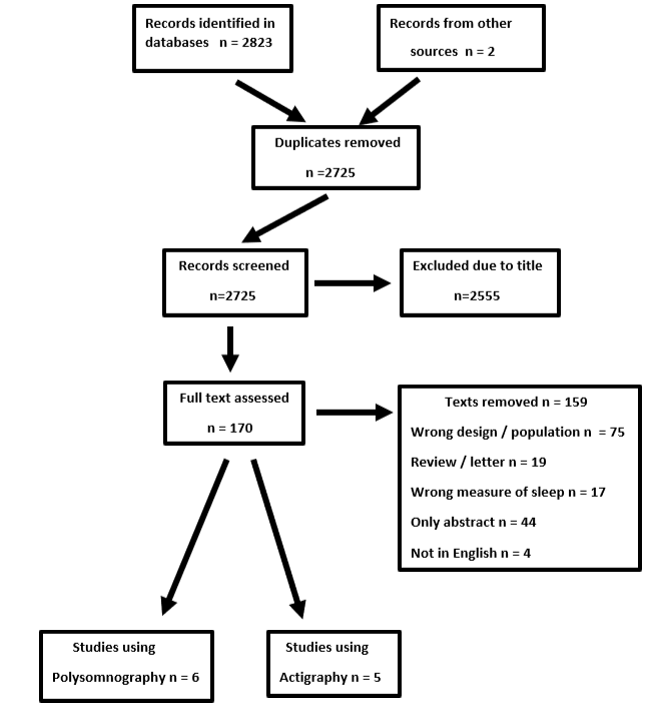
**

**Figure S1 Selection of studies.**

**Quality and bias**

The Study Quality Assessment Tools^2^ of the US National Institute of Health was used,  to assess the quality of the studies.  Twelve questions were answered and agreed by two assessors,  producing yes / no answers. 1) Was the question clearly stated ?; 2) Were selection criteria clearly described? ; 3) Were the participants representative of the population of interest? ; 4)  Were all eligible participants enrolled? ; 5) Was the sample size sufficient to give confidence in the findings? ; 6)  Was the intervention clearly described and delivered consistently? ; 7)  Were the outcomes prespecified, clearly defined, valid, reliable, and assessed consistently? ; 8)  Were the people assessing the outcomes blinded to the participants' interventions? ; 9) Was the loss to follow-up  20% or less? Were any lost accounted for? ; 10) Did statistical methods examine changes from before to after the intervention? Were p values provided for the pre-to-post changes? ; 11) Were outcome measures taken multiple times before the intervention, and multiple times after the intervention? ; 12) If conducted at a group level did the statistical analysis use individual-level data to determine effects at the group level?  There was a maximum score of 12 points:  studies, rated 0–4, were labelled poor, 5–8 fair and 9–12 good.

The analysis of Fanelli^3^was used also to identify additional potential sources of bias. Various questions were examined. Are smaller studies more likely to report larger effects? Do earlier studies report more extreme effects? Are significant papers more likely to be published from laboratories in the US – a pressure said to reflect the need to publish to advance your career^3^. Were studies funded by industry, or did having co-authors employed by industry make it more likely to report significant findings? Funnel plots were examined to look for evidence of publication bias.

**Statistical analysis**

The analysis was performed using Cochrane RevMan  5.4.1 ^4^.  The effect size was established using standardized mean differences with 95% confidence intervals, of the differences between the means of those with higher and lower percentages of energy from carbohydrate.  A random-effect model was used, as the possibility could not be excluded that the extent of any effect varied in different studies, reflecting differences in the percentage of energy as carbohydrate.  With a random effect model, tau-squared indicates whether the size of the effect varies between studies^5^. The I^2^ statistics indicate the degree that variation between studies was due to heterogeneity rather than chance ^6^.  The possibility of publication bias was considered by examining funnel plots.

**RESULTS**

**Polysomnography  and carbohydrate consumption**

**
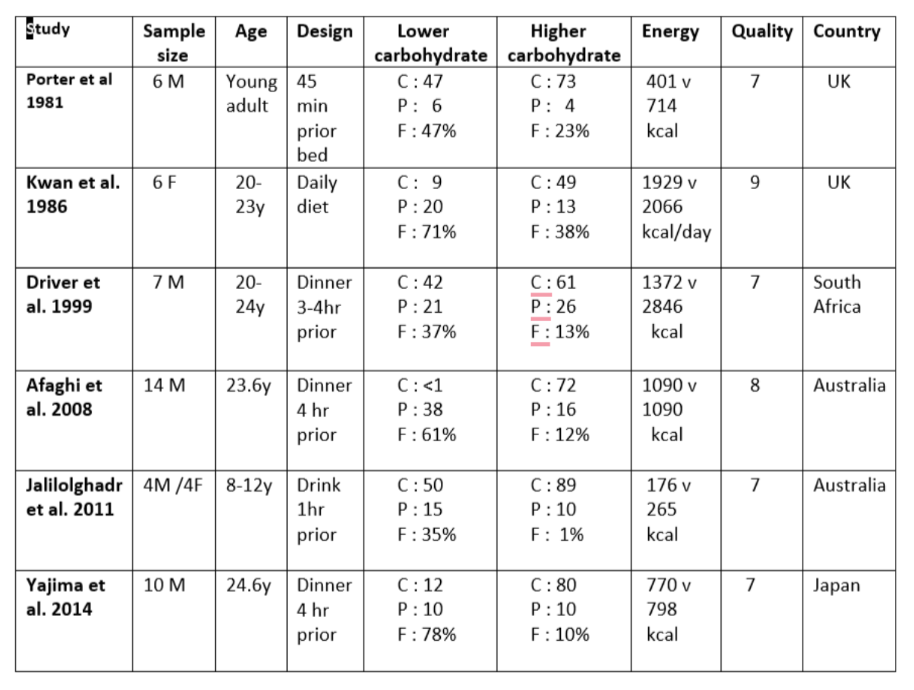
**

**Table S1    Studies using polysomnography to compare the effects of different levels of energy as carbohydrate**

The data were from six studies ^7-12^. The percentages of energy from macronutrients were reported as carbohydrate C; protein P; fat F.   M = male; F = female. The quality of studies was based on the Study Quality Assessment Tool ^3^ of the US National Institute of Health, where a score 0-4 was poor, 5-8 fair and 9-12 good.

The effects were examined, of consuming higher or lower calories as carbohydrate,  prior to recording the sleep pattern using polysomnography.   Table S1 outlines six studies that compared consuming food that contained a higher rather than lower level of carbohydrate. Then, overnight, the architecture of sleep was established (Figure S2).


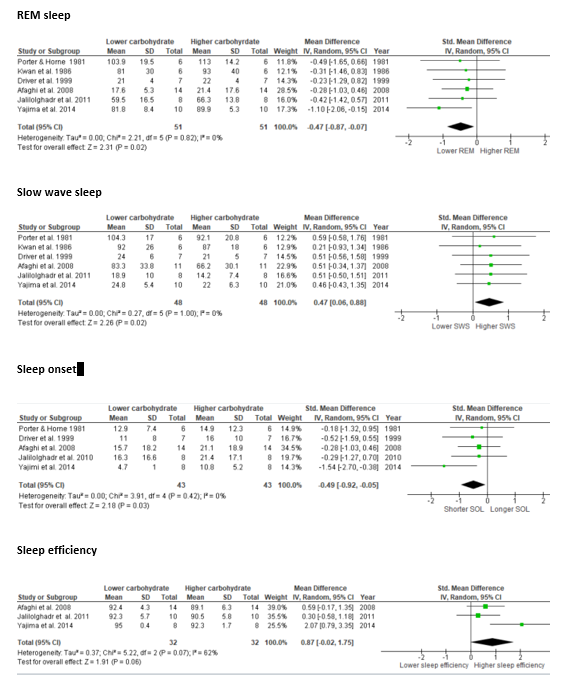


**Figure S2  The influence of differences in carbohydrate consumption on sleep**

When REM was examined the outcome reached significance (RR of -0.47 (95% CI -0.87 to -0.07), p=0.02); I^2^0%): a lower consumption of carbohydrate was associated with a shorter duration of REM.  With SWS again the outcome was significant (RR of 0.47 (95% CI 0.06 to 0.88),  p=0.02; I^2^0%);  a lower intake of carbohydrate was associated with a greater incidence of SWS.

When sleep onset was considered, a lower intake of carbohydrate was associated with a shorter time before falling asleep (RR of -0.49 (95% CI -0.92 to -0.05),  p=0.03; I^2^0%).    There was similarly a trend for better sleep efficiency to be associated with lower intake of carbohydrate (RR of 0.87 (95% CI -0.02 to 1.76),  p=0.07; I^2^0%).

When the study quality was considered all studies had a similar profile (Table S1).  All fell into the overall ‘fair category’, having almost identical profiles.  The limitations reflected small sample sizes (6-14 subjects); four out of five studies only used male young adults questioning the generality of the findings; no study reported sleep analysis being carried out double-blind.

Considering the criteria for bias found by Fanelli^3^, there was no tendency for earlier studies to have produced larger and more significant findings, or to be associated with particular countries (four different countries).  Only one study had any industrial association and that was with a manufacturer of beds rather than food.

**Actigraphy and carbohydrate consumption**

Five studies were identified (Supplementary Table S2) that had compared sleep following the consumption of different percentages of energy as carbohydrate. The groups varied in size from 100 to 188 subjects.

These studies, using actigraphy, failed to produce any significant influence (Figure S3): Sleep onset (SMD = 0.00 (95% CI -0.20 to 0.20), p=1,00; I^2^ 0%); Sleep Efficiency (SMD = 0.17 (95% CI -0.03 to 0.37), p=0.10; I^2^ 0%); Sleep duration (SMD = 0.06 (95% CI -0.22 to 0.34), p=0.67; I^2^ 0%). As four out of five samples were associated with the same laboratory, the possibility exists that the findings reflect common experimental characteristics, although the studies were well designed.

| **Study** | **Sample** | **Age** | **Design** | **Lower carbohydrate** | **Higher**  **carbohydrate** | **Energy** | **Quality** | **Country** |
| --- | --- | --- | --- | --- | --- | --- | --- | --- |
| **Lindseth**  **et al. 2011 (1)** | 44? | 20.6y | 4 day  diet | C : 22  P : 22  F : 56% | C : 50  P : 15  F : 35% | Individual  baseline  intake | 10 | USA |
| **Lindseth**  **et al. 2011 (2)** | 44? | 20.6y | 4 day  diet | C : 22  P : 56  F : 22% | C : 56  P : 22  F : 22% | Individual  baseline  intake | 10 | USA |
| **Misra**  **et al.**  **2015** | 21M  35F | 1.5y | Follow  on formula | C : 34  P : 22  F : 44% | C : 66  P : 10  F : 24% | 421 v 462 kcal  / 100g powder | 9 | Malaysia |
| **Lindseth**  **&**  **Murray 2016 (1)** | 32M  4F | 20.9y | 4 day  diet | C : 25  P : 10  F : 65% | C : 50  P : 15  F : 35% | Individual  baseline  intake | 10 | USA |
| **Lindseth**  **&**  **Murray 2016 (2)** | 32M  4F | 20.9y | 4 day  diet | C : 40  P : 45  F : 15% | C : 80  P : 10  F : 10% | Individual  baseline  intake | 10 | USA |

**Table S2 Studies using actigraphy to compare effects of different levels of energy as carbohydrate**

Three studies were examined although two produced two data sets ^13-15^. Both the Lindseth papers compared four meals, that for analysis were split into two groups of two meals. The pairings were chosen based a preliminary examination of the findings so that the meal with the greatest and least effect on sleep were not compared. The intention was to bias against obtaining significant differences. The percentage of energy from carbohydrate C; protein P; fat F. M = male; F = female. The quality of studies was based on the Study Quality Assessment Tool (2020) of the US National Institute of Health, where a score 0-4 was poor, 5-8 fair and 9-12 good. ? – gender not reported.

**
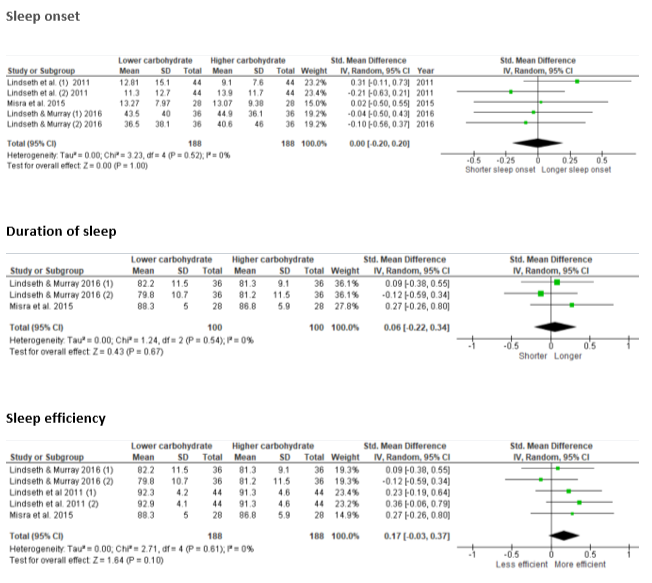
**

**Figure S3 The consumption of different percentage of energy as carbohydrate and measures using actigraphy**

**Discussion**

A significant finding was that meals lower in carbohydrate were associated with more SWS and less REM (Figure S2). Table S1 lists the energy coming from macro-nutrients in this polysomnographic analysis. Particularly relevant is the percentage of energy coming from protein, as only when this is well below 10% is the level of tryptophan in the blood raised^16^. Since the effect on sleep did not depend on having low levels of protein (Table S1), this strongly suggested that influencing the level of tryptophan was not the mechanism.

It seems probable that we should not be treating polysomnograpy and actigraphy as generically measuring sleep; the outcomes reflect different parameters. For example, comparing meals varying in the amount of carbohydrate, failed to find significant differences when using actigraphy (Figure S3), whereas it did with polysomnography (Figure S2).

Further discussion can be found in the main paper.

**REFERENCES**

# 1)Page MJ, McKenzie JE, Bossuyt PM, Boutron I, Hoffmann TC, Mulrow CD, et al.

# The PRISMA 2020 statement: an updated guideline for reporting systematic reviews.

# BMJ 2021;372:n71.

# 2)Study Quality Assessment Tools. 2 Available online:

# https:[//www.nhlbi.nih.gov/health-topics/studyquality-assessment-tools](https://www.nhlbi.nih.gov/health-topics/study-quality-assessment-tools) (Accessed

# 10^th^ December, 2020).

# 3)Fanelli D, Costas R, Ioannidis JPA. Meta-assessment of bias in science. Proc Nat Acad

# Sci. 2017; 114: 3714-3719.

4) Review Manager Web . Version 5.4.1. The Cochrane Collaboration, 2020.

5)  Borenstein M, Hedges LV, Higgins JPT et al.  Introduction to Meta‐Analysis**.**2009**,**John Wiley & Sons.

6)     Higgins JPT, Thompson SG, Deeks JJ, et al.  Measuring inconsistency in meta-analyses.  *Brit. Med. J.* 2003;  327: 557.

7)       Porter JM, Horne JA.  Bed-time food supplements and sleep: effects of different

carbohydrate levels. *Electroencephalogr  Clin Neurophysiol.* 1981; 51: 426–433.

8)     Kwan RM, Thomas S, Mir MA. Effects of a low carbohydrate isoenergetic diet on

sleep behavior and pulmonary functions in healthy female adult

humans. *J Nutr.* 1986; 116: 2393–2402.

9)     Driver HS, Shulman I, Baker FC, et al.  Energy content of the evening meal alters nocturnal

body temperature but not sleep. *Physiol Behav*. 1999; 68: 17–23.

10)  Afaghi A, O’Connor H, Chow CM.  Acute effects of the very low carbohydrate diet on

sleep indices. *Nutr Neurosci*. 2008; 11: 146–54.

11)  Jalilolghadr S,  Afaghi A,  O'Connor H, et al.  Effect of low and high glycaemic index drink on sleep pattern in children.   *J Pakistan Med Assoc*. 2011; 61: 533-536.

12) Yajima K, Seya T, Iwayama K, et al. Effects of nutrient composition of dinner on sleep architecture and energy metabolism during sleep*. J Nutr Sci*Vitaminol. 2014; 60: 114–121.

 13) Lindseth G, Lindseth P, Thompson M. 2013. Nutritional Effects on Sleep *West J Nurs*

*Res.* 2013; 35: 497-513.

14) Lindseth A G, Murray A. Dietary Macronutrients and Sleep. *West J Nurs Res* 2016; 38:

938-958.

15) Misra S, Khor GL, Mitchell P, Haque S, Benton D. A pilot study to determine the short-

term effects of milk with differing glycaemic properties on sleep among toddlers: a

randomised controlled trial. *BMC Pediatr*. 2015; 15: Article 79.

16) Benton D, Donohoe RT. The effects of nutrients on mood.

*Public Health Nutr.* 1999; 2: 403–409.
